# The Price of Mercy: Comment to the Paper Entitled “Prevention of Beta Thalassemia In Northern Israel - A Cost-Benefit Analysis” by Koren et Al. recently published in Mediterranean Journal of Hematology and Infectious Diseases

**DOI:** 10.4084/MJHID.2014.022

**Published:** 2014-03-10

**Authors:** Piero C. Giordano, Eliezer Rachmilewitz

**Affiliations:** 1 Emeritus Associated Professor, Clinical Biochemical Molecular Geneticist. Hemoglobinopathies Laboratory; Dpt. of Human and Clinical Genetics; Leiden University Medical Center, Holon, Israel.; 2 Department of Hematology; The Edith Wolfson Medical Center, Holon, Israel.

Hemoglobinopathies (HBP) are the most frequent genetic recessive diseases in human. These conditions results from mutations in either the alpha or beta globin genes, causing structural modifications (abnormal hemoglobins) or expression defects (thalassemias) that will affect the formation of the hemoglobin molecules.^1^

It is estimated that due to malaria selection, about 4% of the world population, are carriers of significant HBP traits particularly in tropical and subtropical regions of the old world, in the Mediterranean basin and south east Asia. Today however, due to massive migrations, carriers of sickle cell disease (SCD) and thalassemia are spread all over the world and need diagnostics and prevention. Prevention of severe recessive diseases is because in every pregnancy where the couple has clinically relevant mutation, there is a 25% chance that the fetus will receive the mutated genes from both parents. The clinical outcome in such cases is a severe disease such as thalassemia major (TM) or SCD that, in spite of extensive and expensive treatment will only aggravate until premature death.

When a full match hystocompatible donor is available, it is possible in some cases to “cure” thalassemia by bone marrow transplantation (BMT). However, even if successful, BMT may not repair the existing organ and tissue damage and may result in major complications such as infections and graft vs. host disease. Because of the complexity and the costs, BMT is not available in many countries. Therefore the majority of the patients will be treated with supportive therapies. For SCD it consists of frequent hospitalizations and opioids during crises and management of the many chronic and acute complications. For severe TM frequent blood transfusions (one or two units every 2–3 weeks) are needed lifelong!^2^ Expensive chelation therapy must be applied to get rid of the excess of iron (200mg in every blood unit). Without constant chelation the patients will develop iron overload with severe damage to major organs and die mostly due to heart failure.

If possible, the best treatment for all diseases is prevention. Different kinds of secondary and primary prevention are applicable to HBP. Morbidity (secondary) prevention is the kind of prevention offered by newborn screening (NBS). Diagnosis at birth allows for both TM and SCD early genotype/phenotype correlation, prognosis and tailored/preventive treatment to be offered at the beginning of the symptoms, which will be in most cases around 5–6 months of age. Moreover, parents who had a sick child can be identified by NBS, and can be counseled and consequently make a retrospective reproductive choice for the next pregnancy. Similarly, parents at risk who had a carrier of the disease (50%) will be recognized and may have a prospective primary prevention in the future. However, couples at risk who had a “non-carrier” (25%) child will remain unaware until the first affected child is born.

It will be clear that NBS will not reduce the incidence of the disease and that screening at an earlier stage is the best HBP prevention strategy. In addition, it has been shown that mainly due to inadequate counseling, primary prevention after NBS is not effective and that screening should be offered early in pregnancy also in non-endemic countries.^3^ Screening before pregnancy allows more prevention options, from adapting partner choice to remaining childless, having gamets donation, pre-implantation diagnostics (PGD) or, the most common, prenatal diagnosis (PD). However, screening before pregnancy is not customary in non-endemic countries while screening early in pregnancy is socially the most rational alternative. Moreover, screening early in pregnancy does not stigmatize the female partner (common in some cultures when screening is carried out before marriage) and it involves both parents in the process of choosing to accept or not to have a severely affected child. The disadvantage is however that screening early in pregnancy leaves as only prevention option PD and medical abortion. Deciding to interrupt a pregnancy, even if in an early stage and knowing to bear a severely affected fetus, is an emotional process for most couples involving their moral feelings and/or religious believes. Religious leaders have different views on this matter, some may reject medical abortion regardless of the situation and other may consider it as an act of mercy. Practically, the standard PD procedure consists of extracting and analyzing fetal DNA obtained from chorion villi collected by the 10^th^–12^th^ week of gestation or from the amniotic fluid 2 weeks later but non-invasive technologies are upcoming.^4^ The risk of fetal loss is in good hands below 1%, without risk to the mother, giving her the chance to give birth to another (healthy) child in the future.

Primary prevention needs the support of public health authorities and when it comes to investing money in prevention, medical and moral arguments may have a relative impact and one has to show that primary prevention is not only an act of mercy sparing huge suffering to patients and families but that it is an intervention that reduces the costs of public health as well.

Koren et al. approach this economical matter in a proper way, showing that there are major differences in the expenses involved in prevention compared to those involved in treatment of TM.^5^ The expenses for running prevention program in Northern Israel during one year (2011) was about 415.000 $, while the annual cost of basic treatment of one patient including blood transfusions and iron chelation was around 40.000$ and for estimated life expectancy of 50 years, about 2.000.000 $. Moreover, these numbers do not account for the treatment of the various complications, unlike the calculated expenses for blood transfusions, iron chelation etc. that are more or less the same in every patient. However, even if the complications are not the same for every patient, according to the data presented in table 5 of Koren’s paper, around 200.000 $ can be added to the total cost for life expectancy of 2.000.000 $ over 50 years. By enlarge, the costs of treating thalassemia patients is more or less in the same ball park in Israel when compared to the Western countries like U.K, U.S.A., Canada and Italy, while in Thailand they are less, but still much higher than the cost of prevention. The authors could also have taken into consideration that these patients, even when they reach adult life, are often not self-supporting and are social security dependent.

Although it seems so obvious that implementation of screening program and prenatal diagnosis will save a lot of money in addition to sparing huge suffering to patients and their families (up to 76000.000 $ in 10 years due to prevention of the birth of 45 affected newborns in Northern Israel), there are still several problems that have to be addressed. Some countries have implemented compulsory screening before marriage (Cyprus, Iran, UAE). Although efficient, obligatory procedures will encounter many objections in other countries. Therefore offering the possibility of carrier screening early in pregnancy, eventually coupled to rhesus screening which is offered in most countries at the national level and well accepted, seems to be the most efficient non-compulsory approach.^3^,^6^,^7^

In conclusion, since the expenses for treatment of HBP are considerably higher when compared to prevention, even if they vary in different countries, the paper of Koren et al. emphasizes to the medical community and health authorities that the advantages of implementation of a preventive policy which includes prenatal diagnosis, is not only humane but is also economically justified.
